# A Comparison of Microstructure and Microhardness Properties of IN718 Fabricated via Powder- and Wire-Fed Laser-Directed Energy Deposition

**DOI:** 10.3390/ma16031129

**Published:** 2023-01-28

**Authors:** Nandana Menon, Brady A. Sawyer, Cory D. Jamieson, Edward W. Reutzel, Amrita Basak

**Affiliations:** 1 Department of Mechanical Engineering, The Pennsylvania State University, University Park, PA 16802, USA; 2 Applied Research Laboratory, The Pennsylvania State University, University Park, PA 16802, USA

**Keywords:** laser directed energy deposition, IN718, microstructure, microhardness properties

## Abstract

The objective of this work is to compare the microstructure and microhardness properties of IN718 deposited by both powder- and wire-fed laser-directed energy deposition (L-DED) processes. The powder-fed L-DED is carried out on an Optomec LENS^®^ system while the wire-fed L-DED is performed in an in-house custom-built system. Several single-layer single-track specimens are fabricated using different combinations of process parameters to down-select the optimal process parameters for both systems. The finalized parameters are, thereafter, used to build thin-wall specimens having identical designs. The specimens are characterized using optical and electron microscopy as well as microhardness measurements. The results demonstrate that the powder-fed specimen, built using optimal process parameters, does not exhibit any distortion. On the contrary, the wire-fed specimen, built with optimal process parameters, show lesser porosity. Differences in elemental segregation are also detected in the two specimens. For example, nitrides and carbides are observed in the wire-fed specimen but not in the powder-fed specimen. The microhardness measurements reveal the powder-fed specimen has higher microhardness values compared to the wire-fed specimen. These results can be used to fabricate parts with sequential powder and wire deposition to achieve biomimetic structures of varying microstructure and microhardness properties.

## 1. Introduction

In recent years, laser additive manufacturing (LAM) has gained unprecedented attention in the fabrication of complex metal parts and geometries that are otherwise challenging to produce with conventional methods. A popular subset of LAM is laser-directed energy deposition or L-DED. L-DED is extensively used to fabricate functionally-graded parts and large structures, and to repair legacy components [1]. Nickel-based superalloys such as IN718 have been processed substantially using L-DED because of their superior weldability. IN718 is widely used in propulsion and power generation industries because of its high-temperature capability and strength. It is a precipitation-hardened alloy, predominantly consisting of Fe, Nb, and Mo with trace amounts of Al and Ti. The corrosion resistance arises from the presence of Ni and Cr that crystallize as a face-centered cubic (FCC) γ phase. The strengthening phase is the metastable body-centered tetragonal Ni${}_{3}$Nb which comprises the hardening precipitates $\gamma^{\prime \prime }$. The intermetallic $\gamma^{\prime }$ phase, consisting of Ni${}_{3}$(Ti, Al) simple cubic crystal precipitates, has a lower degree of hardening compared to $\gamma^{\prime \prime }$ [2]. The extreme toughness and work-hardening characteristics pose challenges in machining IN718 [3]. L-DED is, therefore, a viable alternative to fabricating near-net-shape IN718 parts.

L-DED can be conducted using both wire and powder as feedstock. Powder deposition has better controllability compared to wire feeding. Therefore, more complex structures can be built through powder-fed L-DED [4]. Metal powders can, however, absorb moisture, oxygen, and other impurities from the surroundings due to their large surface areas. These impurities can change the overall composition of the powder impacting its processability. On the contrary, wire allows for better process efficiency while being safer to use. Powder-fed L-DED of IN718 has been widely explored in the open literature. Previous studies have investigated the effects of process parameters on the macro and microstructural characteristics of the printed components. Zhao et al. [5] studied the microstructures and mechanical properties of L-DED specimens built using gas-atomized (GA) IN718 powder. The study concludes that the specimens fabricated using GA powders are susceptible to porosities and cracks. Parametric studies were conducted to optimize the process parameters for fabricating single-layered thin-wall structures of IN718 by Jinoop et al. [6,7]. The effect of processing parameters such as the laser power, scan velocity, working distance, and initial substrate temperature on the microhardness, fusion zone morphology, and microstructure was studied by Kistler et al. [8]. The study revealed that altering the process parameters was beneficial in producing parts with tailored material properties. Li et al. [9] investigated the variation in microstructures and mechanical properties that occur at different locations in the powder-fed L-DED specimens. In the bottom and middle regions of the specimens, δ, $\gamma^{\prime \prime }$, and $\gamma^{\prime }$ phases were observed while the top of the specimens was devoid of these precipitates. The hardness was also observed to vary throughout the specimens, remaining similar in the bottom and middle regions of the specimens, but decreasing near the top of the specimens.

As opposed to powder-fed L-DED, wire-fed L-DED is rather nascent. Zhang et al. [10] studied the microstructures and mechanical properties of multi-layer and multi-track IN718 specimens built using a continuous wave fiber laser with filler wire addition. The heat-affected zone was reported to consist of γ matrix, globular Ni${}_{3}$Nb-δ precipitates, Nb-rich MC type primary carbides and carbonitrides, as well as strengthening phases, $\gamma^{\prime }$ and $\gamma^{\prime \prime }$. Brittle Laves phase particles were also observed in the interdendritic region. Arrizubieta et al. [11] evaluated and compared the efficiency and mechanical properties of IN718 components built using both powder- and wire-fed L-DED. The number of layers required to achieve the desired deposit height was fewer in wire-fed L-DED than powder-fed L-DED due to the higher material deposition rate in the former. No significant difference was observed in the mechanical properties and hardness, however, the powder-fed L-DED specimens were found to be more brittle. Bambach et al. [12,13] investigated the microstructures of IN718 cuboids built using powder, cold wire, and hot wire L-DED via a six-beam direct diode laser system. All specimens were observed to have dendritic microstructures dominated by columnar grains. Additionally, Laves phases were found in the interdendritic region. The hot-wire specimens contained Ti- and Nb-rich MC type carbides, whereas the cold-wire specimens showed no signs of either. The powder-fed L-DED specimens contained Al and Ti nitrides, which were found in neither cold- nor hot-wire specimens. Local recrystallization zones consisting of fine equiaxed grains were observed only in the hot-wire specimen owing to the low energy input. Table 1 summarizes the key findings obtained from the literature involving L-DED of IN718.

To that end, this paper compares the microstructures and microhardness properties of the IN718 specimens fabricated via both powder- and wire-fed L-DED. Identical specimen designs are adopted for both types of deposition. The powder-fed L-DED is carried out on an Optomec LENS^®^ system while the wire-fed L-DED is performed in an in-house custom-built system. Several single-layer single-track specimens are fabricated using different combinations of process parameters to down-select the optimal process parameters for both systems. The finalized parameters are, thereafter, used to build thin-wall specimens. The specimens are characterized using optical and electron microscopy as well as microhardness measurements. On the macroscale level, warpage or distortion is observed in the wire-fed specimen. However, this specimen shows lesser porosity compared to its powder-fed counterpart. Differences in elemental segregation are detected in the two specimens. Nitride and carbide precipitates are seen in the wire-fed specimen while oxides are present in both the powder- and wire-fed specimens. Additionally, the powder-fed specimen is found to have higher microhardness values compared to the wire-fed specimen.

The paper is organized into four sections as follows: The current introductory section is followed by the experimental details that outline feedstock characterization and discusses the L-DED systems used in the present study. This section is followed by the results section that discusses the findings obtained from optical microscopy (OM), scanning electron microscopy (SEM), energy dispersive X-ray spectroscopy (EDS), and microhardness measurements. Finally, concluding remarks are provided in the last section of the paper.

## 2. Experimental Details

### 2.1. Materials: Virgin Powder and Wire

The IN718 powder (product name: Osprey^®^ Alloy 718-AM), used in this study for powder-fed L-DED, is acquired from Sandvik Osprey (Neath, United Kingdom). This powder is produced using a gas atomization process in an Argon environment. The wire feedstock (product name: Techalloy^®^ 718) is obtained from Lincoln Electric (Cleveland, Ohio, United States). The chemical composition of the powder and wire feedstock is reported in Table 2 [15,16].

**Figure 1:**
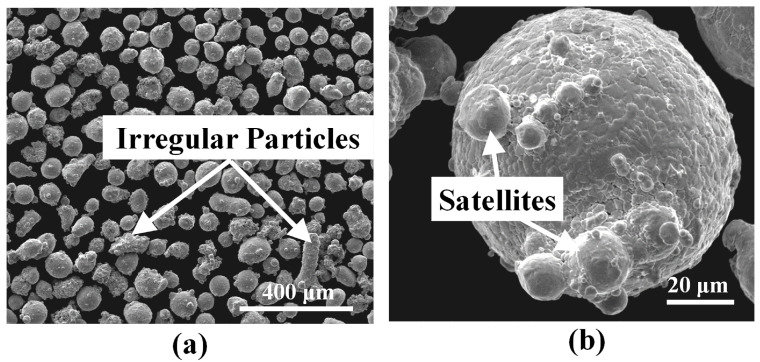
(**a**) SEM image of the virgin IN718 powder. (**b**) A high-magnification SEM image showing one of the larger particles with satellites attached to it.

**Figure 2:**
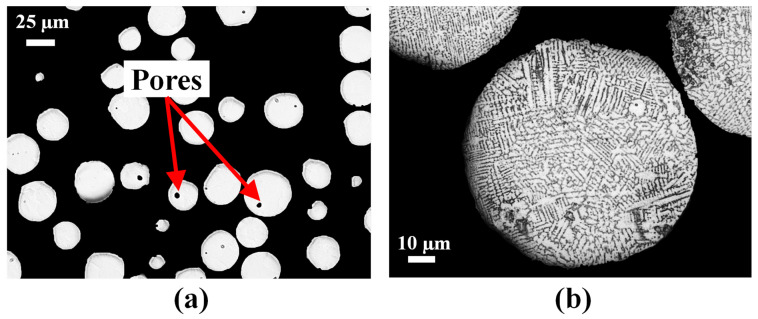
Cross-sectional OM image of the virgin IN718 powder: (**a**) polished, revealing the pores present inside the powder particles and (**b**) etched, highlighting the dendritic microstructure.

The morphology of the virgin IN718 powder is analyzed using SEM. The IN718 powder particles are found to be mostly spherical as shown in Figure 1a but contain several irregularly shaped particles. Some particles are found to have satellites attached to them as shown in Figure 1b. To study the particle size distribution, sieve analysis is carried out. The results are tabulated in Table 3. Hausner ratio values below 1.25 have been linked with good flowability properties in the existing literature [17]. The powder used in this study shows a Hausner ratio of 1.23 implying it has good flowability. The powder is mounted into Bakelite and mirror polished using standard metallography techniques, which are further delineated in Section 2.2. Figure 2a shows the polished cross-sectional image of the virgin IN718 powder as revealed by OM. The powder particles exhibit internal porosity that is typical of GA powders [5]. The mounted powder is further etched. The etched cross-section of a representative powder particle is shown in Figure 2b depicting dendritic microstructure.

**Figure 3:**
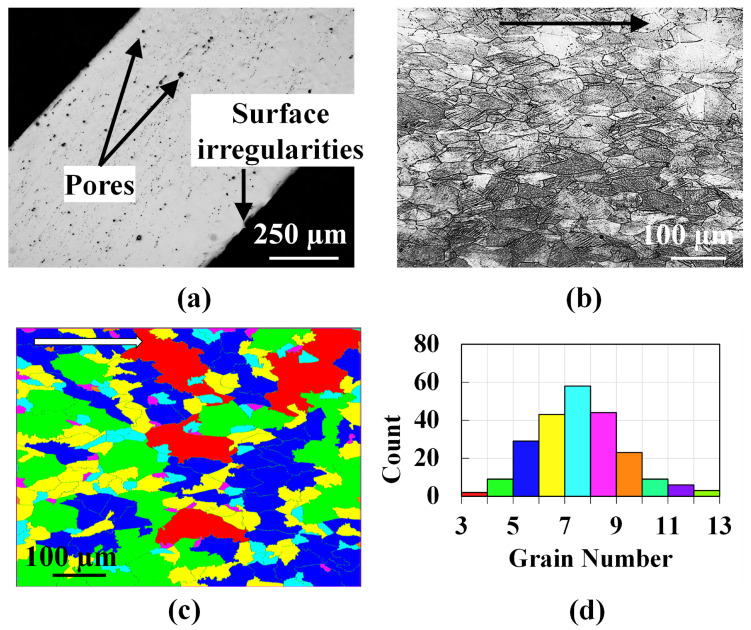
(**a**) Cross-sectional OM image of the polished IN718 wire revealing internal and surface pores. (**b**) Cross-sectional image of the etched IN718 wire depicting the grains. The black arrow in (**b**) indicates the drawing direction. (**c**) A representative etched cross-section analyzed using LAS X Grain Analysis module and color coded according to the grain number. The white arrow in (**c**) indicates the drawing direction. (**d**) The histogram of grain number.

OM investigation is performed on the IN718 wire as well. The polished cross-section of the IN718 wire reveals internal pores of varying diameters, as observed in Figure 3a. Surface irregularities are also present. The average diameter of the wire is found to be 0.87 mm. The polished cross-section of the IN718 wire is etched. The grains are found to elongate along the drawing direction, as depicted in Figure 3b. The etched image is then analyzed using the LAS X’s Grain Expert module of the Leica OM software. Standard test method such as ASTM E112 [18] is applied to determine the average grain size which is expressed as grain number. Figure 3c shows the grains color coded according to their grain numbers. The distribution of the grain number is shown in Figure 3d indicating a Gaussian one.

### 2.2. Manufacturing Methods

**Figure 4:**
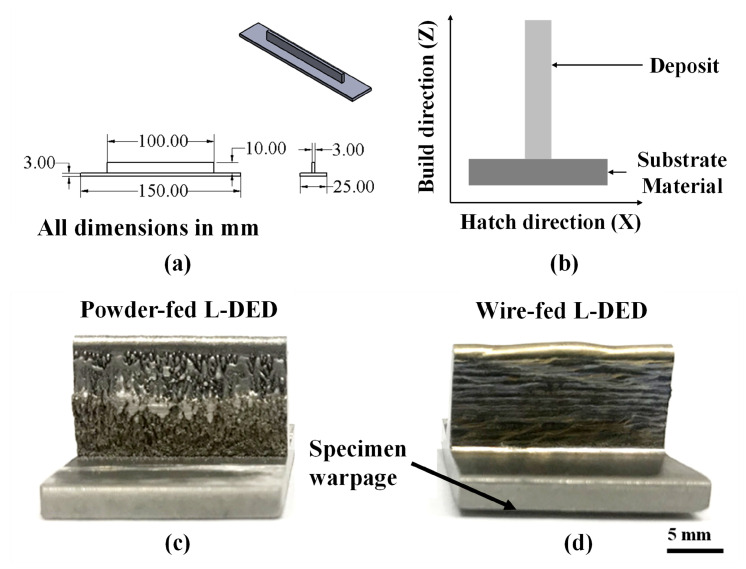
(**a**) Geometry of the thin-wall specimen. (**b**) A schematic showing a representative cross-section for microstructure investigation and microhardness measurements. Images of the physical specimens: (**c**) powder-fed and (**d**) wire-fed.

The geometry of the thin-wall specimens is shown in Figure 4a. The microstructure investigation and the microhardness measurements are performed on a representative cross-section as shown in Figure 4b. The powder-fed thin-wall specimen is fabricated using Optomec LENS^®^ MR-7 consisting of an IPG Photonics YRL 500 W ytterbium fiber laser, under an Argon gas environment. This system has a 3 Axis CNC Control System and a working envelope of 30 cm × 30 cm × 30 cm. The laser power level can be upgraded to 1 kW to produce near-net-shape parts. The wire-fed specimen is fabricated using a custom-built L-DED system utilizing 3rd-generation AeroMet high-deposition capabilities. This system employs an IPG Photonics YRL 12 kW ytterbium fiber laser in a customizable build volume with rectilinear builds that can span 100 cm × 30 cm × 45 cm. The system is equipped with custom cladding head along with water-cooled optics and an Argon gas enclosure. The system is located at the Applied Research Laboratory, Penn State—University Park.

Several single-layer single-track experiments are performed to determine the optimal process parameters for both systems [19,20]. Compared to the powder-fed specimen, the wire-fed specimen shows a smooth and shiny metallic surface as depicted in Figure 4c,d. The powder-fed specimen is devoid of any warping. However, the wire-fed specimen shows slight warping. The finalized parameters are listed in Table 4. The hatch spacing is maintained to have an overlap of 33%. Thermal management is incorporated for the wire-fed L-DED specimen by manually measuring the build-plate temperature using a thermocouple probe and, thereafter, manually starting the toolpath. Both the thin-wall specimens are fabricated on HAYNES^®^ 718 AMS 5596 substrates.

### 2.3. Characterization Methods

Specimens for microstructural investigation are sectioned using a band saw and thereafter mounted in Bakelite. The mounted specimens are wet grounded using SiC paper; starting with 120 grit and progressively moving to finer grit size, up to 800 grit. The specimens are then polished to mirror finish using 3 μm and 1 μm diamond solutions. Finally, the specimens are polished using a 0.5 μm colloidal alumina suspension. The polished specimens are then etched using Kalling’s 2 reagent (50 mL HCl, 50 mL H${}_{2}$O, and 10.0 g CuSO${}_{4}$) for ∼20 s to reveal the microstructure. OM imaging is completed using a Leica DM 6M optical microscope. SEM investigations are carried out on two systems—the Apreo S and the Verios G4, the latter providing a shorter working distance. The SEM systems are equipped with an Oxford Instruments Aztec Energy EDX system that is used for the elemental analysis. Vickers microhardness measurements are carried out using a Qness 60.

### 2.4. Materials: Used Powder

**Figure 5:**
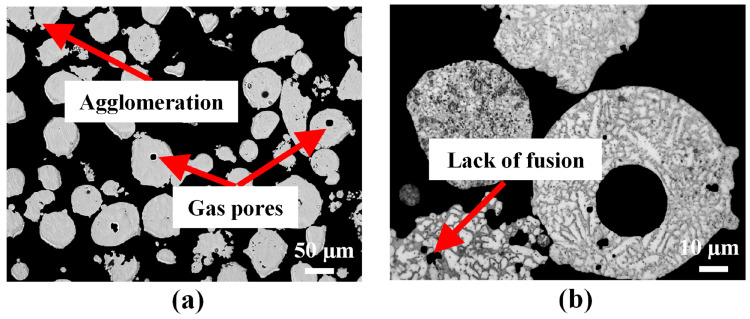
(**a**) Cross-sectional OM image of the used IN718 powder revealing the pores. (**b**) Cross-sectional OM image of the etched powder highlighting the microdendrites.

Used powder from the powder-fed L-DED system is collected, and their cross-sections are examined to characterize and evaluate the level of degradation that could affect their possible reuse. Figure 5 shows the cross-sectional OM images of the used powder. A visual inspection of the used powder shows the presence of a large number of irregularly shaped particles with agglomerations or satellites. The polished cross-sections shown in Figure 5a reveal the pores in the used powder. Figure 5b shows the coarser dendritic structure prevalent in the used powder particles. The results demonstrate how the virgin powder loses its homogeneous microstruture due to cyclic heating during L-DED [21].

## 3. Results and Discussion

### 3.1. Investigation of the Deposits Using OM

**Figure 6:**
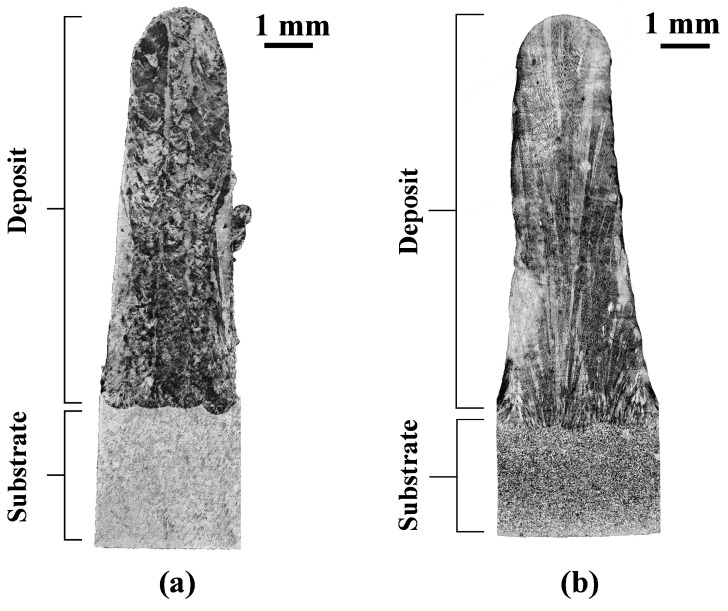
OM image of the etched cross-section of (**a**) the powder-fed L-DED specimen and (**b**) the wire-fed L-DED specimen.

**Figure 7:**
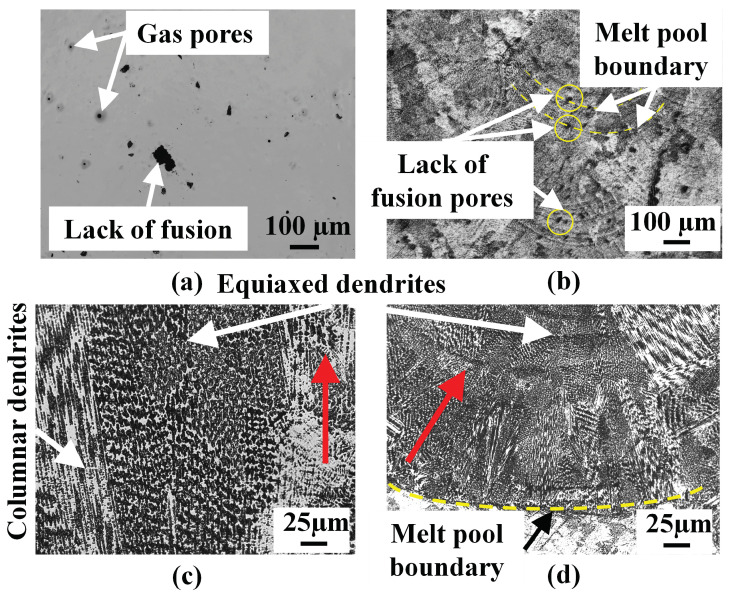
(**a**) OM image highlighting gas and lack of fusion pores. (**b**) Etched OM images of the powder-fed L-DED specimen highlighting the melt pool boundaries and lack of fusion pores. Microstructure revealing both columnar and equiaxed dendrites: (**c**) at the middle of the specimen and (**d**) in the melt pool of the first layer near the substrate. The vertical red arrow in (**c**) indicates the build direction. The red arrow in (**d**) represents the growth direction of the columnar dendrites.

The OM images of the etched longitudinal cross-section corroborating to Figure 4b of the powder- and wire-fed L-DED specimens are shown in Figure 6a and Figure 6b, respectively. Excellent metallurgical bond exists between the deposits and substrates. The powder-fed specimen shows several pores along the build direction. These lack of fusion and gas pores are highlighted in Figure 7a. Pores of highly spherical morphology are indicative of gas entrapment. This is also corroborated by their dimensions which conform to those reported in the open literature [22]. The presence of such gas porosities may be attributed to the inert gas entrapped in the powder particles, as seen in Figure 2a, that ultimately remains in the melt pool due to rapid cooling rates [5]. Irregular-shaped lack of fusion pores, typically arising at the boundary of adjacent tracks and layers, as seen in Figure 7b, are observed due to insufficient energy density. This indicates that, while the selected process parameter may have been optimal for single line deposits, the final geometry of the part also has an influence on the defects due to the process parameters. Overall, porosity is observed to decrease along the build direction due to an increase in the degree of remelting with the addition of layers.

During L-DED, the deposit is subjected to cyclic thermal stresses due to the subsequent accumulation of layers. The microstructural features of the deposit, such as the grain size, are typically influenced by the thermal history during the deposition process. The as-built microstructure is primarily dependent on the combined effects of temperature gradient (*G*) and the solidification rate (*R*). The G/R ratio determines the growth of columnar vs. equiaxed grains. A higher G/R ratio favors the formation of columnar grains while equiaxed grains are formed at lower thermal gradients and higher cooling rates [23]. Conduction dominates the region in contact with the substrate. On the contrary, convection dominates the central and top regions of the as-built deposit. This complex heat transfer occurring during an L-DED process is manifested in the form of spatially evolving microstructures throughout the build [24,25].

Upon inspecting the powder-fed specimen, columnar structures are observed to dominate till about mid-height, as seen in Figure 7c, owing to higher thermal gradients, with regions of finer equiaxed grains present between the columnar grains. Such behavior has also been reported in the literature [26,27]. The size of columnar grains is observed to be larger in the middle of the deposit height compared to the bottom layers. With the addition of subsequent layers, the temperature increases, thereby, decreasing the thermal gradient. Hence, the top layers are subjected to more homogeneous cooling rates resulting in the equiaxed microstructure. In the powder-fed L-DED specimen, the thermal gradient is highest at the melt pool boundary, for the first layer, due to the adjacent substrate that acts as a heat sink. Due to the large undercooling that results from this temperature gradient, nucleation occurs at this region giving rise to fine columnar grains that grow perpendicular to the melt pool boundary (Figure 7d). The columnar dendrites converge toward the center confirming the presence of large thermal gradient perpendicular to the melt pool boundary in the mushy zone. At the center of the melt pool, equiaxed grains suppress the formation of columnar grains giving rise to columnar-to-equiaxed transition due to higher G/R ratios [28].

**Figure 8:**
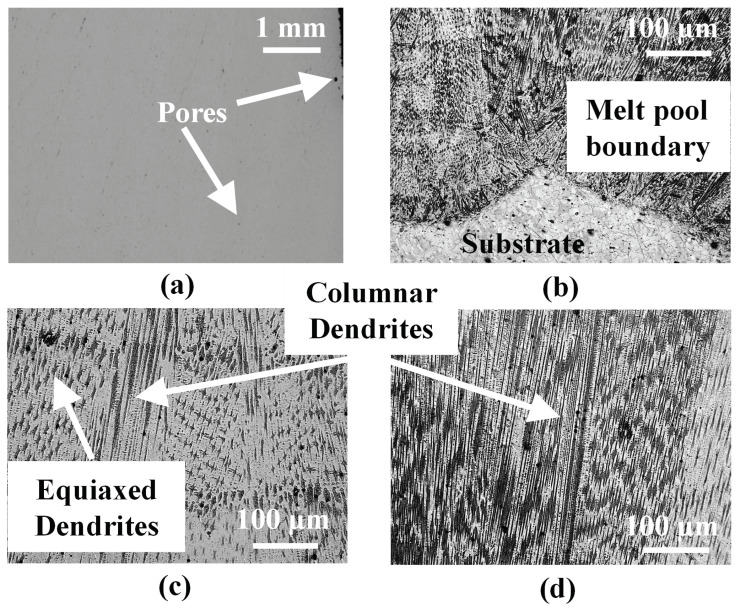
(**a**) Unetched cross-sectional OM image of wire-fed L-DED specimen. Etched OM image of the wire-fed L-DED specimen at: (**b**) the first layer of the deposit and substrate interface. This location has both columnar and equiaxed dendrites with some of the columnar dendrites extending to the deposit-substrate interface; (**c**) near the central region of the deposit showing both columnar and equiaxed dendrites; and (**d**) near the top of the deposit showing columnar dendrites.

The wire-fed L-DED specimen is analyzed like the powder-fed L-DED specimen. The cross-section of the wire-fed L-DED specimen is observed to have fewer pores. Shown in Figure 8a are pores observed in the wire-fed DED specimen. Process related pores are observed, which may be attributed to the shielding gas. Compared to the powder-fed specimen, the wire-fed specimen is built with higher laser power and lower scan speed. Because of a higher energy density input, the G/R ratio is high and large enough for columnar dendrites to extend over the entire deposit. Shown in Figure 8b is the first layer of the deposit adjacent to the substrate. At this location, mostly columnar dendrites are observed with a very few equiaxed dendrites at the centre that are perhaps suppressed by the overlapping of successive melt pools. In the center of the deposit (Figure 8c), both columnar and equiaxed dendrites are observed, with bands of equiaxed dendrites between sections of columnar dendrites. Again, the columnar grains observed at mid-height are coarser compared to those at the bottom layers. The columnar dendrites do not grow parallel to each other as indicated by the white arrows. After the first few layers of the deposit, these columnar dendrites begin to follow a more uniform orientation and are oriented in the direction of the build, indicating the flow of heat in the center of the deposit is perpendicular to the substrate [14] as shown in Figure 8d.

### 3.2. Characterization of Microstructures Using SEM

**Figure 9:**
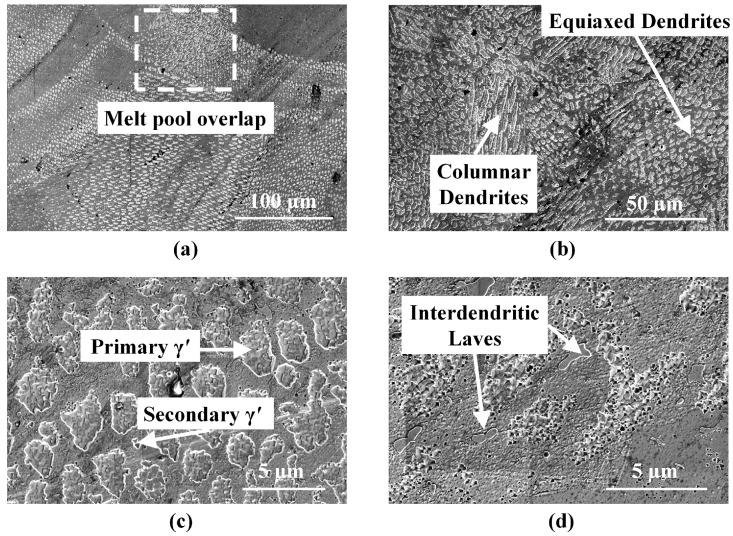
SEM images of the powder-fed L-DED specimen: (**a**) at the bottom of the deposit showing overlapping melt pool boundary; (**b**) at the middle of the deposit where both equiaxed and columnar dendrites are observed; and (**c**) at the top of the build where larger $\gamma^{\prime }$ precipitates are observed due to a decrease in the cooling rate. (**d**) A representative SEM image of the deposit where Laves phases are observed in the interdentritic regions.

Images of the powder-fed L-DED specimen reveal dendrites with short arm spacing throughout the build with any kind of higher order arm growth completely suppressed. The primary dendrite arm spacing is found to be in the range of 2.65 µm. In some cases, as seen in Figure 9a, the direction of dendrite growth is rotated 90 degrees when crossing a melt pool boundary and at the regions where successive tracks overlap. Figure 9b shows a magnified view of the dendritic structure highlighting both equiaxed and columnar dendrites, such as those obserevd in Figure 7c.

**Figure 10:**
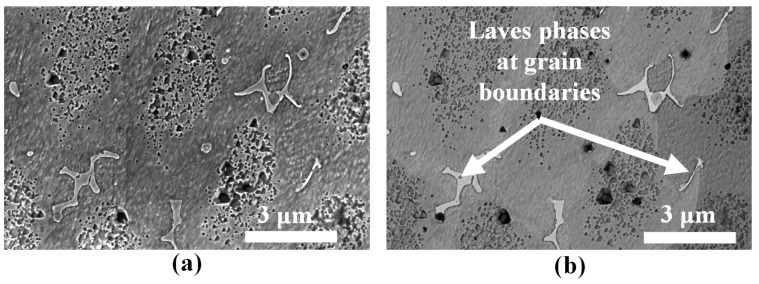
(**a**) SEM image of a representative region in the powder-fed specimen showing Laves phases morphology at the grain boundaries. (**b**) The corresponding backscatter image of the region. Here the Laves phase appear lighter since they are composed of a heavier element (Nb).

Like other nickel-based superalloys, the as-built IN718 deposits mainly comprise of austenitic FCC γ phases and secondary $\gamma^{\prime }$ phases. The dendritic structure is composed of solid solution of γ phase while primary and secondary $\gamma^{\prime }$ precipitates of varying dimensions are dispersed in the γ matrix, as seen in Figure 9c. The total volume of $\gamma^{\prime }$ precipitates decreases with increasing cooling rate. The cooling rate decreases along the build direction with the portion of the deposit closest to the substrate being subjected to higher cooling rates. Consequently, finer cuboidal precipitates are observed in regions closer to the substrate. Nb-rich Laves phases are observed in Figure 9d. The brittle intermetallic Laves phase is widely distributed in the matrix with majority located in the inter-dendritic zones. Some Laves phases are also present at grain boundaries as depicted in Figure 10a,b. The light-colored structures in the backscatter electron image Figure 10b indicate the presence of a heavier element (Nb) compared to the rest of the matrix that is richer in a lighter element (Ni).

**Figure 11:**
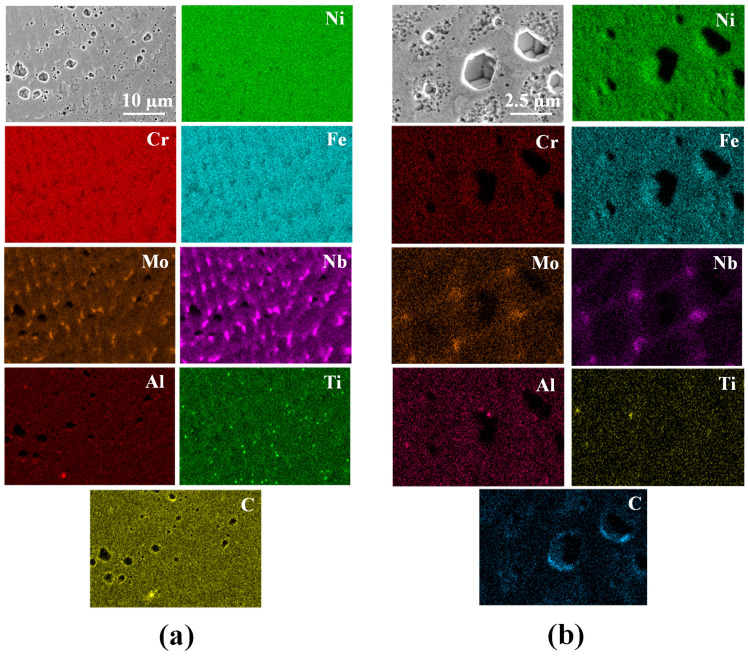
EDS map of a deposit region near the (**a**) bottom and (**b**) top of the powder-fed L-DED specimen highlighting the secondary phases.

**Figure 12:**
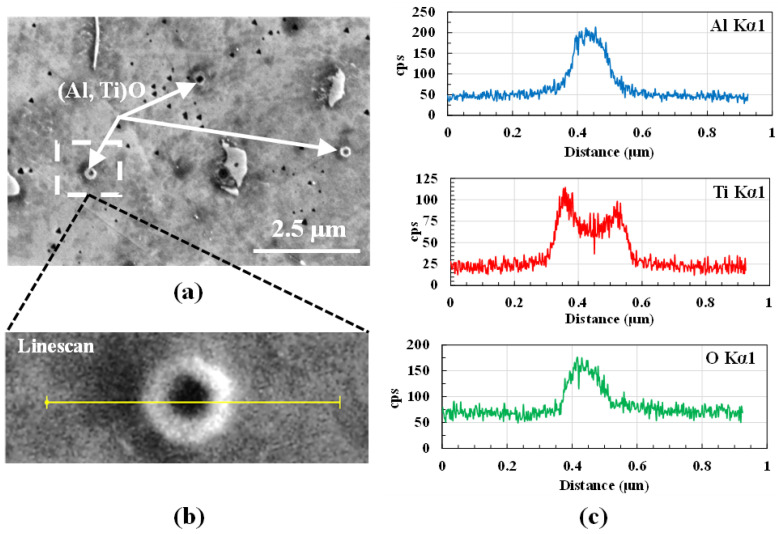
(**a**) SEM image of Al and Ti precipitates in the powder-fed specimen. (**b**) Zoomed image of the region where EDS line scans are taken. (**c**) Line scan profiles of Al, Ti, and O.

Figure 11 illustrates the EDS map of a representative region in the deposit. The maps highlight the segregation of Ti and Al in the interdendritic region and dendritic core. The results demonstrate the presence of γ which, in the dendrite cores enriched in Al, has a detrimental effect on the properties. On the contrary, the interdendritic precipitates enriched in Ti are advantageous [29]. Regions with local enrichment of Mo and Nb at the expense of Cr are observed to coincide with the white regions in the SEM images and, hence, confirm the presence of Laves phases in the deposit. Randomly distributed smaller particles are further analyzed and confirmed using EDS. Al${}_{2}$O${}_{3}$ and TiN are commonly occurring non-metallic particles in the processing of IN718 [30]. Figure 12 shows a small circular particle of ∼20 nm diameter, which upon implementing a linescan shows local enrichment of Al and O. The formation of these spherical Al${}_{2}$O${}_{3}$ particles can be attributed to the increased affinity of Al to O, mostly as a result of exposure of the feedstock powder prior to use. Despite the process being carried out in an inert gas environment, a thin layer of oxide tends to form on the surface of the powder particles. The dip in the Ti scan between 0.35 µm and 0.55 µm reveals that, presumably, the Al${}_{2}$O${}_{3}$ particle is encapsulated at the center of a TiN particle [31].

**Figure 13:**
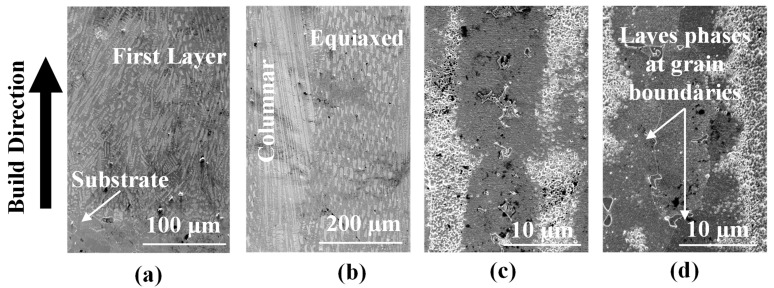
SEM images of the wire-fed L-DED specimen showing (**a**) the first layer adjacent to the substrate and (**b**) the middle region of the specimen where equiaxed and columnar dendrites are observed. SEM images of the (**c**) interdendritic Laves phases and (**d**) Laves phases at the grain boundaries.

SEM micrographs of the wire-fed L-DED specimen reveal dendrites varying in characteristics through the build height. The finer dendrites, formed due to higher cooling rates, in the initial layers can be seen in Figure 13a. Figure 13b, imaged near the middle of the specimen, displays columnar and equiaxed dendrites that are highly organized and aligned parallel to the build direction. The mean primary dendritic arm spacing (PDAS) are found to be in the range of 6.727 ± 1.9 µm, 14.7 ± 0.7 µm, and 24.087 ± 3 µm in the bottom, middle, and top sections of the deposit, respectively. The PDAS (in µm) can be used to determine the cooling rate (ε, K/s) using the semi-empirical relation [32]:

(1) $$PDAS=80\varepsilon^{-0.33}$$

Based on Equation (1), the cooling rate is calculated as 1812.96 K/s at the bottom of the deposit. The middle section has a cooling rate of 169.50 K/s, which is an order of magnitude lower. As expected, the lowest cooling rate is observed in the top section, calculated as 37.99 K/s. Some of the columnar dendrites show short secondary arm growth. More prominent secondary arms develop further along the build height with a mean secondary dendritic arm spacing (SDAS) of 5.293 µm. In the middle of the specimen, seen in Figure 13b, columnar and equiaxed dendrites are observed. They seem to appear in uniform orientations. Laves phases are observed throughout the specimen in the interdendritic region and along the grain boundaries as shown in Figure 13c and Figure 13d, respectively.

**Figure 14:**
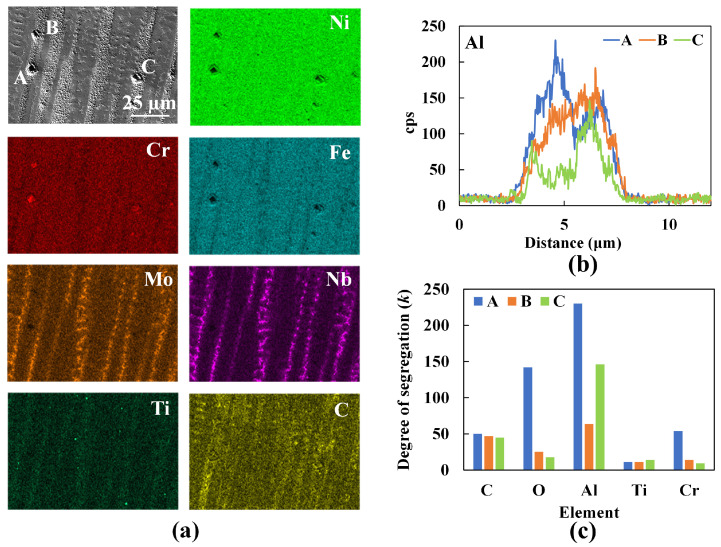
(**a**) EDS map of a representative region in the wire-fed specimen with three precipitates marked as A, B, and C where line scans are obtained. (**b**) EDS line profile of Al across the three marked regions. (**c**) Degree of segregation (*k*) of various alloying elements across the marked regions within the deposit.

**Figure 15:**
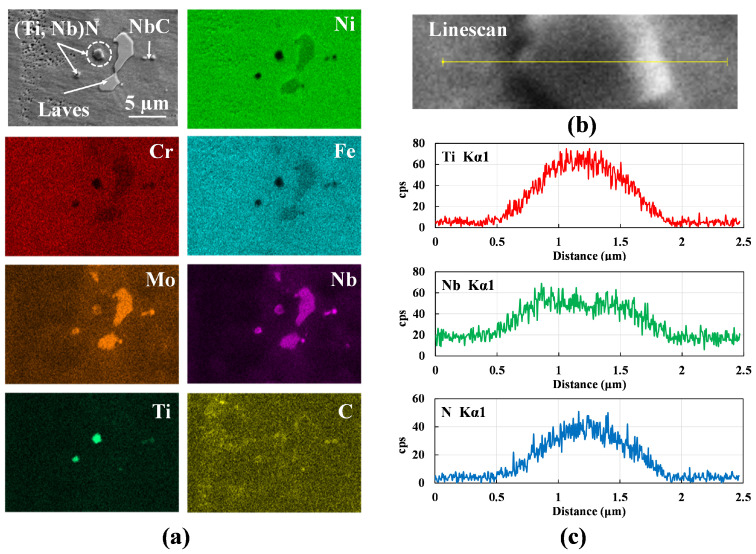
(**a**) EDS map of a representative region within the wire-fed specimen where TiN and NbC precipitates are seen and circled. (**b**) zoomed image of the marked region across which EDS line scan is obtained. (**c**) EDS line profiles of Ti, Nb, and N across the circled precipitate in (**a**) which is zoomed in (**b**).

Figure 14 shows an EDS map of a representative region of the deposit with discrete precipitates labeled as A, B, and C. Elemental line profiles across these precipitates indicate that they are enriched in Al. The maximum and the minimum counts for each element are extracted from their respective line profiles and a segregation parameter, defined as the degree of segregation (*k*) = maximum cps/minimum cps (where cps denotes counts per second) [33], is calculated and plotted in Figure 14b,c. High *k* values for Al and O suggest that the three discrete structures are Al${}_{2}$O${}_{3}$ particles. Small cubic TiN precipitates are seen in the EDS map in Figure 15. TiN precipitates form at a temperature above the liquidus. This precipitate is also enriched in Nb as indicated by the peak observed in the line scan of Nb as shown in Figure 15c. Even a small amount of carbon in the initial feedstock can result in the formation of NbC in the interdendritic regions due to eutectic reactions. NbC precipitates form around 1280–1265 ${}^{{}^{\circ}}$C via reaction L → (γ + NbC). The Laves phase forms eventually at a lower temperature range of 1160–1075 ${}^{{}^{\circ}}$C, via the reaction, L + NbC → (γ + Laves) and L → (γ + Laves) [31]. These Laves phases manifest themselves in the form of islands. The exact temperatures and precipitates can however vary with the composition of the feedstock and processing conditions (e.g., cooling rates).

### 3.3. Analysis of the Deposits Using Microhardness Measurements

**Figure 16:**
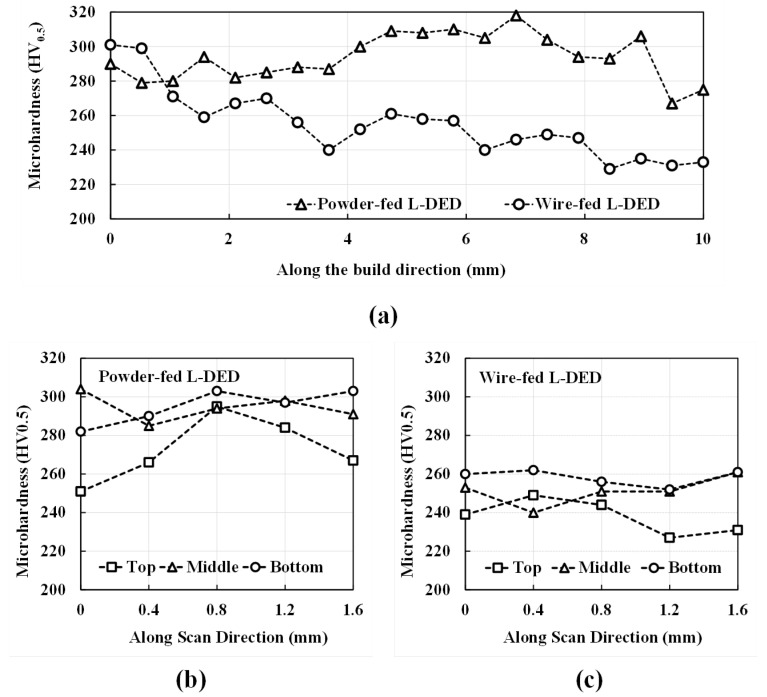
A comparison of the microhardness measurements of powder- and wire-fed specimens: (**a**) along the build direction and along the scan direction at the top, middle, and bottom sections of the (**b**) powder-fed L-DED specimen and (**c**) wire-fed L-DED specimen.

During the L-DED process, due to multiple cycles of cooling and heating, the microhardness of the as-built specimens varies from those manufactured using conventional methods. The microhardness of the L-DED specimens depends on the grain morphology and PDAS, the presence of precipitates, and the alloy composition [34]. Vickers Microhardness is calculated for both powder- and wire-fed L-DED specimens. Indents are made in two directions, i.e., along the build direction and along the hatch direction. Figure 16 shows the microhardness measurements made on the polished deposit region of the powder- and wire-fed L-DED specimens. Microhardness is reported to increase with a decrease in the specific energy [35]. The present results are in agreement with the literature. It can be seen that the powder-fed L-DED specimen, built at a much lower specific energy of 9.72 J/mm${}^{2}$, has a higher microhardness with a mean value of 293.7 HV${}_{0.5}$ measured along the build direction. Since the powder-fed specimen is fabricated at a higher speed and lower power, the overall heat input is lower resulting in a faster cooling rate that refines the microstructure and increases the microhardness. The hardness values also fluctuate along the scan direction due to the inhomogeneous microstructure. For the powder-fed specimen, the average hardness measured in the bottom, middle, and top sections are 295 HV${}_{0.5}$, 294.4 HV${}_{0.5}$, and 272.6 HV${}_{0.5}$, respectively. For the wire-fed L-DED specimen, the average hardness measured in the bottom, middle, and top sections are 258.2 HV${}_{0.5}$, 251.2 HV${}_{0.5}$, and 238 HV${}_{0.5}$, respectively. The hardness values are tabulated in Table 5. Such insight into the microhardness of the as-built IN718 specimens can be used to determine suitable heat treatment strategies. Based on the user requirement, microhardness can be increased by heat treatment applied at low temperatures while the heat treatment applied at high temperatures reduces microhardness [36].

## 4. Conclusions

In this work, thin-wall specimens of IN718 are fabricated using powder-fed and wire-fed L-DED. While the powder-fed specimens are manufactured on a commercial LENS^®^ system, the wire-fed specimens are fabricated using an in-house system. Both types of specimen have identical designs. However, they are built using the optimum process parameters for their respective systems. Microstructural characterization is performed via OM, SEM, and EDS. Additionally, microhardness measurements are carried out.

The powder-fed specimen, built using optimized process parameters, shows no distortion or cracks but has several pores throughout the build. On the contrary, while porosity is minimal, distortion is prevalent in the wire-fed specimen built with optimal process parameters. SEM analysis of both specimens reveals the formation of $\gamma /\gamma^{\prime }$ morphologies in the deposit region. Both specimens show Laves phases distributed along the build, in the interdendritic regions, and at the grain boundaries. While the powder-fed specimen shows no carbides, the wire-fed specimen reveals the presence of NbC precipitates. This may be due to higher C content in the wire feedstock (Table 2). The presence of (Al, Ti) oxides and nitrides are highlighted by EDS maps in the microstructure of both specimens. Hardness measurements, carried out using a microindenter, confirm that the powder-fed specimen possesses a higher Vickers hardness than the wire-fed specimen.

These observations can be gainfully used for determining ideal repair strategies for components via L-DED and/or fabricating functionally graded components. The choice between powder and wire feedstocks should be made based on the following considerations: wire for material efficiency, and if the cost and time of fabrications need to be minimized and powder if defects and distortion need to be minimized, for better process control, and for parts with higher hardness. Future studies include:

- Building and repairing large-scale specimens using powder and wire feedstocks to investigate their mechanical properties such as fatigue.
- Performing heat treatment studies to understand the evolution of microstructures and mechanical properties.
- Fabricating functionally-graded parts via sequential powder and wire depositions as well as powder-wire co-extrusion to achieve biomimetic structures.

## Figures and Tables

**Table 1:** Summary of powder- and wire-fed L-DED for fabricating IN718 specimens.

Feedstock	System	Findings	Ref.
Powder	5 kW ROFIN-SINAR continuous wave CO${}_{2}$ laser, LPM-408 CNC working table, DPSF-1 powder feeding system	Porosities were found in the as-built parts because GA powder was used.	[5]
	ROFIN-SINAR 850 5 kW CO${}_{2}$	γ columnar dendrites and small amount of (γ + Laves) eutectics were seen in the interdendritic regions.	[14]
	2 kW fibre laser, 5 axes, CNC with coaxial nozzle and a twin powder feeder	As-built specimen revealed intermixed dendritic and cellular microstructure.	[6,7]
	Optomec, Inc. LENS^®^ MR-7	Hardness increased from the base metal to the fusion zone. Correlations were observed between process parameters and fusion zone morphology, and microstructure.	[8]
	6 kW semiconductor laser, a three-dimensional CNC working table, DPSF-2 powder feeder with a coaxial nozzle	Variation of $\delta ,\gamma^{\prime },\gamma^{\prime \prime }$ precipitates and microhardness was seen along the build height.	[9]
Wire	IPG Photonics 5 kW continuous wave solid-state Yb-fiber laser system (YLR-5000) equipped with an ABB robot	Precipitates such as $\delta ,\gamma^{\prime },\gamma^{\prime \prime }$, Nb-rich MC type carbides were seen in the lower beads near the interfacial layer.	[10]
Powder vs. Wire	5 axis CNC machine (Alzmetall GX 1000/5-T-LOB), coupled with a 4.5 kW diode lase	Higher deposition rates and efficiency were achieved for wire L-DED.	[11]
Powder vs. Hot Wire vs. Cold Wire	Direct diode laser DED head with a maximum laser power of 1 kW installed on an industrial robot. The process head consisted of six laser beams with the wire heated via resistance heating within the head	The material efficiency for cold wire was 100%. The deposition rate of hot wire was twice than that of the cold wire process. Hot wire specimen showed columnar dendritic and local recrystallized regions. No recrystallized regions were seen in the cold wire and the powder specimens. Laves phases in the interdendritic regions were observed in all three specimens.	[12,13]

**Table 2:** Chemical composition of the powder and wire feedstock [15,16].

Element	Powder Feedstock (wt. %)	Wire Feedstock (wt. %)
Ni + Co	52.55	50.00–55.00
Cr	18.1	17.00–21.00
Nb	4.82	4.75–5.5
Mo	2.9	2.80–3.30
Ti	0.92	0.65–1.15
Al	0.48	0.20–0.80
Si	0.08	0.35 max
Mn	0.05	0.35 max
N	0.04	NA
C	0.014	0.08 max
Cu	0.01	0.30 max
P	0.01	0.015 max
S	0.003	0.015 max
B	0.001	0.006 max
Fe	20.022	Balance
Other	NA	0.50 max

**Table 3:** Summary of powder powder characterization through sieve analysis.

	Calculated Value	STD
Average Flow Rate (g/s)	2.78	0.05
Average Apparent Density (g/cm${}^{3}$)	3.81	0.01
Average Tap Density (g/cm${}^{3}$)	4.67	0.35
Average Hausner Ratio	1.23	0.10
Angle of Repose (deg)	29.00	N/A
Sieve Analysis		
+90 µm	0.20%	
−90 µm + 45 µm	95.80%	
−45 µm	4.00%	

**Table 4:** Process parameters used in the powder- and wire-fed L-DED processes.

	Feedstock	
	Powder	Wire
System	LENS^®^	In-House
Power (W)	350	1460
Spot Size (mm)	1.2	3.5
Travel Speed (in/min)	30	20
Specific Energy (J/mm${}^{2}$)	9.72	57.54
Feed Rate	6.5–6.6 (g/min)	16.19 (in/min)

**Table 5:** Range of microhardness values observed at different locations for powder-fed and wire-fed L-DED specimens.

Along the Build Direction (HV${}_{0.5}$)									
				Min	Max	Std			
			Powder-fed	267	318	13.55			
			Wire-fed	229	301	19.89			
**Along the Scan Direction (HV${}_{0.5}$)**									
	Top			Middle			Bottom		
	Min	Max	Std	Min	Max	Std	Min	Max	Std
Powder-fed	251	295	17.13	285	304	7.16	282	303	9.03
Wire-fed	227	249	9.06	240	261	7.5	252	262	4.15

## Data Availability

The data generated and/or analyzed during the current study are available from the A.B. and C.D.J on reasonable request.
